# Clinical value of grayscale ultrasound combined with real-time shear wave elastography nomogram in risk prediction of thyroid cancer

**DOI:** 10.1186/s12880-023-01099-y

**Published:** 2023-09-12

**Authors:** Tiantian Ren, Mingfei Jiang, Jiawei Wu, Fan Zhang, Chaoxue Zhang

**Affiliations:** 1 Department of Medical Ultrasound, Maanshan People’s Hospital, Hubei Road, Anhui Maanshan, 243032 China; 2 School of Public Health, Southeast University, Hunan Road, Nanjing, Jiangsu 210000 China; 3 Department of Ultrasound, The First Affiliated Hospital of Anhui Medical University, Meishan Road, AnHui Hefei, 230000 China

**Keywords:** Thyroid nodule, Grayscale ultrasound characteristics, Real-time shear wave elastography, Risk prediction model

## Abstract

**Objectives:**

This study constructed a nomogram based on grayscale ultrasound features and real-time shear wave elastography (SWE) parameters to predict thyroid cancer.

**Methods:**

Clinical data of 217 thyroid nodules of 201 patients who underwent grayscale ultrasound, real-time SWE, and thyroid function laboratory examination in Ma’anshan People’s Hospital from January 2019 to December 2020 were retrospectively analyzed. The subjects were divided into a benign nodule group (106 nodules) and a malignant nodule group (111 nodules). The differences in grayscale ultrasound features, quantitative parameters of real-time SWE, and laboratory results of thyroid function between benign and malignant thyroid nodules were analyzed. We used a chi-square test for categorical variables and a *t*-test for continuous variables. Then, the independent risk factors for thyroid cancer were analyzed using multivariate logistic regression. Based on the independent risk factors, a nomogram for predicting thyroid cancer risk was constructed using the RMS package of the R software.

**Results:**

Multivariate logistic regression showed that the grayscale ultrasound features of thyroid nodules were the shape, margin, echogenicity, and echogenic foci of the nodules,the maximum Young’s modulus (SWE-max) of thyroid nodules, and the ratio of thyroid nodule and peripheral gland (SWE-ratio) measured by real-time SWE were independent risk factors for thyroid cancer (all *p* < 0.05), and the other variables had no statistical difference (*p* > 0.05). Based on the shape (OR = 5.160, 95% CI: 2.252–11.825), the margin (OR = 9.647, 95% CI: 2.048–45.443), the echogenicity (OR = 6.512, 95% CI: 1.729–24.524), the echogenic foci (OR = 2.049, 95% CI: 1.118–3.756), and the maximum Young’s modulus (SWE-max) (OR = 1.296, 95% CI: 1.140–1.473), the SWE-ratio (OR = 2.001, 95% CI: 1.403–2.854) of the thyroid nodule to peripheral gland was used to establish the related nomogram prediction model. The bootstrap self-sampling method was used to verify the model. The consistency index (C-index) was 0.979, ROC curve was used to analyze the nomogram scores of all patients, and the AUC of nomogram prediction of thyroid cancer was 0.976, indicating that the nomogram model had high accuracy in the risk prediction of thyroid cancer.

**Conclusions:**

The nomogram model of grayscale ultrasound features combined with SWE parameters can accurately predict thyroid cancer.

## Introduction

Thyroid nodules are a common disease of the endocrine system [1]. Thyroid cancer accounts for only 5-15% of thyroid nodules, but the incidence rate is increasing by 4-6.2% every year [2, 3]. Ultrasound (US) is the preferred imaging method for the diagnosis of thyroid nodules. The grayscale acoustic characteristics of thyroid nodules could be used to characterize thyroid nodules. Scholars from different countries have established their own hierarchical risk assessment system (Thyroid Imaging Reporting and Data System, TI-RADS) according to the grayscale US characteristics of thyroid nodules [4–10]. Despite the widespread use of TI-RADS, the interpretation of thyroid images remains inconsistent, as over 10 different TI-RADS systems are currently used across Chinese hospitals [11]. Additionally, the sensitivity and specificity of these systems in detecting thyroid cancer vary widely, making it challenging to compare data between hospitals.

Shear wave elastography (SWE) is a non-invasive medical imaging technique used to evaluate tissue elasticity by measuring the propagation speed of high-frequency ultrasound waves within tissues. This new imaging technology has improved the detection of various cancer, particularly thyroid cancer [12–15]. In addition, some studies have found a correlation between the extent of the disease on SWE and the serum thyroid-stimulating hormone (TSH) concentrations. However, these studies had conflicting results, and it remains unclear whether TSH promotes or protects against thyroid cancer [16, 17].

In order to address these challenges, we developed a novel machine-learning algorithm to identify the most relevant risk factors for thyroid cancer based on US grayscale characteristics of the nodules, two-dimensional real-time SWE measurements, and patient serum indices. In addition, we also created a nomogram to facilitate the implementation of the machine learning model clinically. Nomograms are statistical models based on logistic regression or Cox regression that can quantitatively and graphically represent prediction indicators and provide a more intuitive evaluation of clinical events [18–21]. This innovative approach could potentially provide a more accurate individualized risk assessment for thyroid nodules than the traditional TI-RADS system.

## Materials and methods

### Study population

We retrospectively analyzed the clinical data of 217 thyroid nodules in 201 patients who were subjected to grayscale US, SWE, and laboratory examination in Maanshan people’s Hospital from January 2019 to December 2020. The inclusion criterion was thyroid nodules confirmed by pathology during operation or cytological puncture. The exclusion criteria were nodules without surgery, poor or unsatisfactory cytological diagnosis of nodules, and atypical or unclear follicular lesions.

### Grayscale US and SWE examinations

The grayscale US and SWE examinations were performed with the same sonography machine (Aixplorer; Supersonic Imagine, Paris, France) with a 5–14 MHz high-frequency linear transducer. The size of thyroid nodules was measured from longitudinal and transverse sections, and the shape of thyroid nodules was evaluated from transverse sections. The shape, margin, echogenicity, echogenic foci, and composition of thyroid nodules were observed and recorded. The shape was divided into wider than tall and taller than wide. The margin was divided into smooth, ill-defined, lobulated or irregular, and extrathyroidal extension. The echogenicity of the solid portion was classified as hyperechogenicity or isoechogenicity, hypoechogenicity, or very hypoechogenicity. The hyperechoic foci were divided into non-calcification or large comet-tail artifacts, macrocalcifications, peripheral (rim) calcifications, punctate echogenic foci, or mixed calcifications. The internal compositions were categorized as cystic or almost completely cystic, spongiform, mixed cystic and solid, and solid or almost completely solid.

SWE was used to examine the thyroid nodules. The maximum value (SWE-max), minimum value (SWE-min), mean value (SWE-mean), and the ratio of nodules to glands (SWE-ratio) were measured from longitudinal sections. During the measurements, the diameter of the Q-box was 2 mm, and the unit was kPa. The average of each value was calculated from measurements taken three times.

All images are collected and read by two radiologists with more than 10 years of experience.

### Laboratory examination

The thyroid-related biochemical indexes were measured using a DPP-800 biochemical automatic analyzer (Roche, Germany). The free triiodothyronine (FT3), serum-free thyroxine (FT4), total triiodoethyl-3 (T3), total thyroxine-4 (T4), and TSH were also measured.

### Statistical analysis

The R software (v3.6.1) was used for statistical analyses. Chi-square tests were used for categorical variables, and *t***-**tests were used for numerical variables. Multivariate logistic regression was used to analyze the influencing factors in thyroid nodule malignant incidence. The independent risk factors were analyzed by R software’s RMS package, and the nomogram of thyroid cancer risk was constructed. The test level was 0.05, and *p* < 0.05 meant that the difference was statistically significant.

### Diagnostic performance and validation of the nomogram

In the nomogram, a vertical line was made on the score axis at the top of the coordinate axis of the independent variable to obtain the corresponding score; the score of each independent variable was summed up to obtain the total score. The bootstrap self-sampling method was used to evaluate the results of the nomogram, and the consistency index (C-index) was obtained: C-index = 0.50–0.70 was low accuracy, 0.70–0.90 was moderate accuracy, and > 0.90 was high accuracy.

## Results

### Comparison of baseline data, US parameters, and laboratory tests for the prediction of thyroid malignancy

The results showed 28 males and 78 females with a mean age ± standard deviation (SD) of 51. 90 ± 10.13 years (range: 18–71 years) with benign nodules. There were 34 males and 77 females with a mean age ± SD of 44.28 ± 46 years (range: 21–65 years) with malignant nodules. The mean nodule size was 17.9 ± 1.16 mm (range: 3.8-69.9 mm).There were significant differences in the shape, margin, echogenicity of the solid portion, hyperechoic foci, internal composition of grayscale US nodule features, SWE-max, SWE-min, SWE-mean, and SWE-ratio between benign and malignant nodules (*p* < 0.05). There was no significant difference in other variables between the two groups (p > 0.05) (Table 1).

**Table 1:** Comparison of baseline data, US parameters, and laboratory tests for the prediction of thyroid malignancy

Variable		Benign nodule (*n* = 106)	Malignant nodule (*n* = 111)	*t*/*χ*^*2*^	*p*
Age (year)		51.90 ± 10.13	44.28 ± 9.46	5.73	< 0.001
Gender(male %)		28(26.42)	34(30.63)	0.47	0.492
Shape	Wider-than-tall	92(86.79)	54(48.65)	35.84	< 0.001
	Taller-than-wide	14(13.21)	57(51.35)		
Margin	Smooth	85(80.19)	25(22.52)	79.69	< 0.001
	Ill-defined	12(11.32)	19(17.12)		
	Lobulated or irregular	5(4.72)	54(48.65)		
	Extrathyroidal extension	4(3.77)	13(11.71)		
Echogenicity	Anechoic	0	0	-	< 0.001
	Hyperechoic or isoechoic	61(57.55)	8(7.21)		
	Hypoechoic	35(33.02)	72(64.86)		
	Very hypoechoic	10(9.43)	31(27.93)		
Echogenic Foci	Nonecalcification or Large comet-tail artifacts	84(79.25)	40(36.04)	50.36	< 0.001
	Macrocalcificatins	13(12.26)	19(17.12)		
	Peripheral (rim) calcifications	2(1.89)	1(0.90)		
	Punctate echogenic foci	7(6.60)	51(45.95)		
Composition	Cystic or almost completely cystic	0	0	-	< 0.001
	Spongiform	3(2.83)	0		
	Mixed cystic and solid	30(28.30)	1(0.90)		
	Solid or almost completely solid	73(68.87)	110(99.10)		
SWE	Mean, kPa	29.37 ± 12.92	45.98 ± 26.65	-5.80	< 0.001
	Min, kPa	23.01 ± 11.45	35.38 ± 20.11	-5.54	< 0.001
	Max, kPa	35.32 ± 14.64	54.49 ± 33.12	-5.47	< 0.001
	Ratio	2.79 ± 1.74	2.24 ± 1.53	-2.48	0.014
FT3, IU/ml		4.76 ± 0.67	4.85 ± 0.76	-0.88	0.379
FT4, IU/ml		15.91 ± 2.89	16.87 ± 2.90	-2.47	0.014
T3, IU/ml		1.66 ± 0.31	1.68 ± 0.30	-0.43	0.667
T4, IU/ml		103.00 ± 25.75	103.90 ± 20.54	-0.27	0.785
TSH, IU/ml		3.41 ± 2.59	2.85 ± 2.37	1.64	0.103

### Analysis of influencing factors of thyroid cancer

The indexes with statistical significance in Table 1 were taken as independent variables and analyzed by multivariate logistic regression. The results showed that the shape, margin, echogenicity, echogenic foci, and elastic measurement values (SWE-max and SWE-ratio) were independent risk factors for thyroid cancer (Table 2).

**Table 2:** The shape, margin, echogenicity, echogenic foci, and elastic measurement values (SWE-max and SWE-ratio) were independent risk factors for thyroid cancer

Variables	β	SE	X2	*p*	OR
**Intercept**	-17.03	3.53	23.30	< 0.001	
**Shape**	2.27	0.79	8.21	0.004	9.647
**Margin**	1.64	0.42	15.04	0.001	5.160
**Echogenicity**	1.87	0.68	7.67	0.006	6.512
**Echogenic foci**	0.71	0.31	5.38	0.020	2.049
**SWE-Max**	0.26	0.07	15.70	< 0.001	1.296
**SWE-Ratio**	0.69	0.18	14.66	0.001	2.001

### Nomogram for predicting the risk of thyroid cancer

**Fig. 1:**
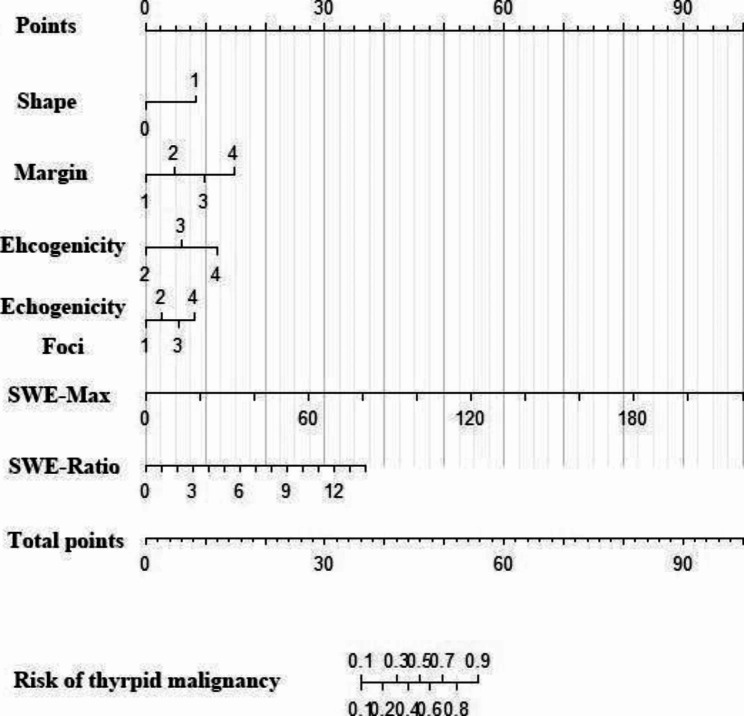
The nomogram was constructed with the shape, margin, echogenicity, and echogenic foci of thyroid nodule and the elastic measurement values SWE-max and SWE-ratio as dependent variables. Shape (1: wider-than-tall; 2: taller-than-wide), margin (1: smooth, 2: ill-defined, 3: lobulated or irregular, 4: extrathyroidal extension), echogenicity (2: isoechoic, 3: hypoechoic, 4: very hypoechoic), echogenic foci (1: non-calcification or large comet-tail artifacts, 2: macrocalcifications, 3: peripheral (rim) calcifications, 4: punctate echogenic foci). To use, find the corresponding value in the ‘Total points’ score, and make a vertical line down to the ‘Risk of thyroid malignancy’ to determine the risk of thyroid nodules

**Fig. 2:**
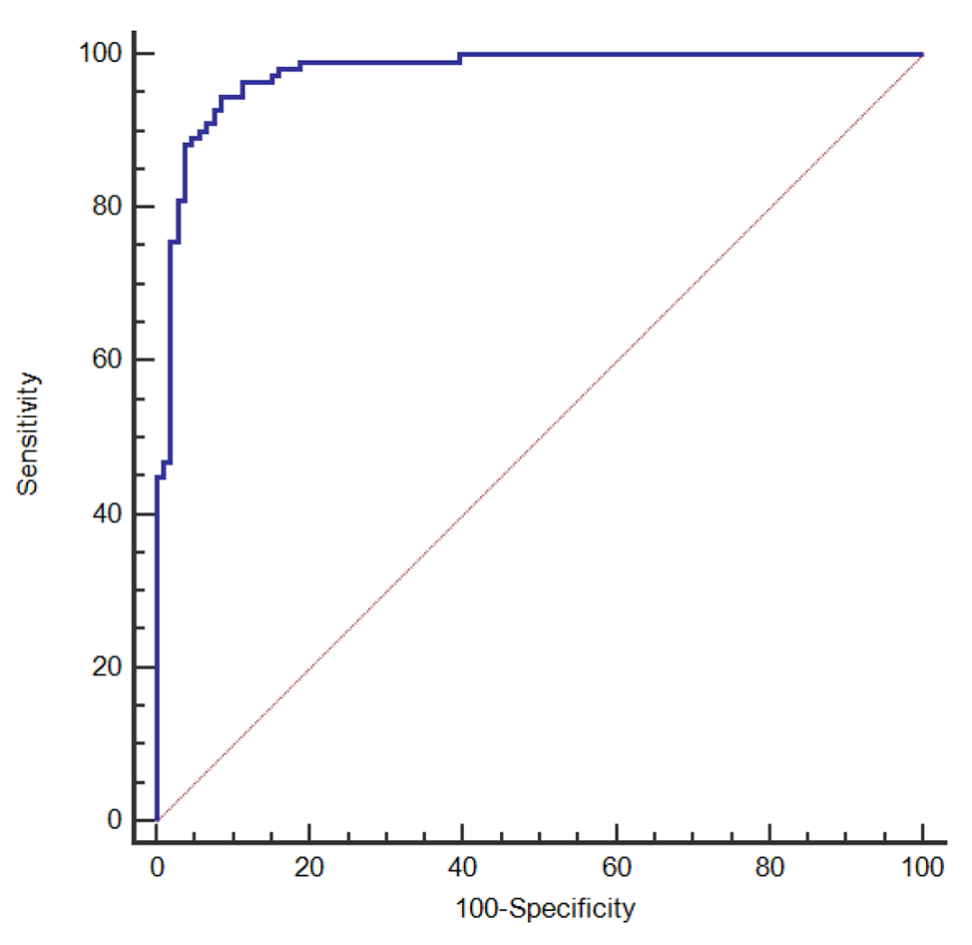
ROC curve was used to analyze the nomogram scores of all patients, and the AUC of nomogram prediction of thyroid cancer was 0.976

The nomogram was constructed with shape, margin, echogenicity, echogenic foci, and the elastic measurement values (SWE-max and SWE-ratio) as dependent variables. After bootstrap self-sampling, the C-index was 0.979, with high accuracy. In clinical application, the possible risk of thyroid cancer can be predicted using the nomogram. See Figs. 1 and 2.

### An example of the proposed nomogram in use for the risk of thyroid malignancy

**Fig. 3:**
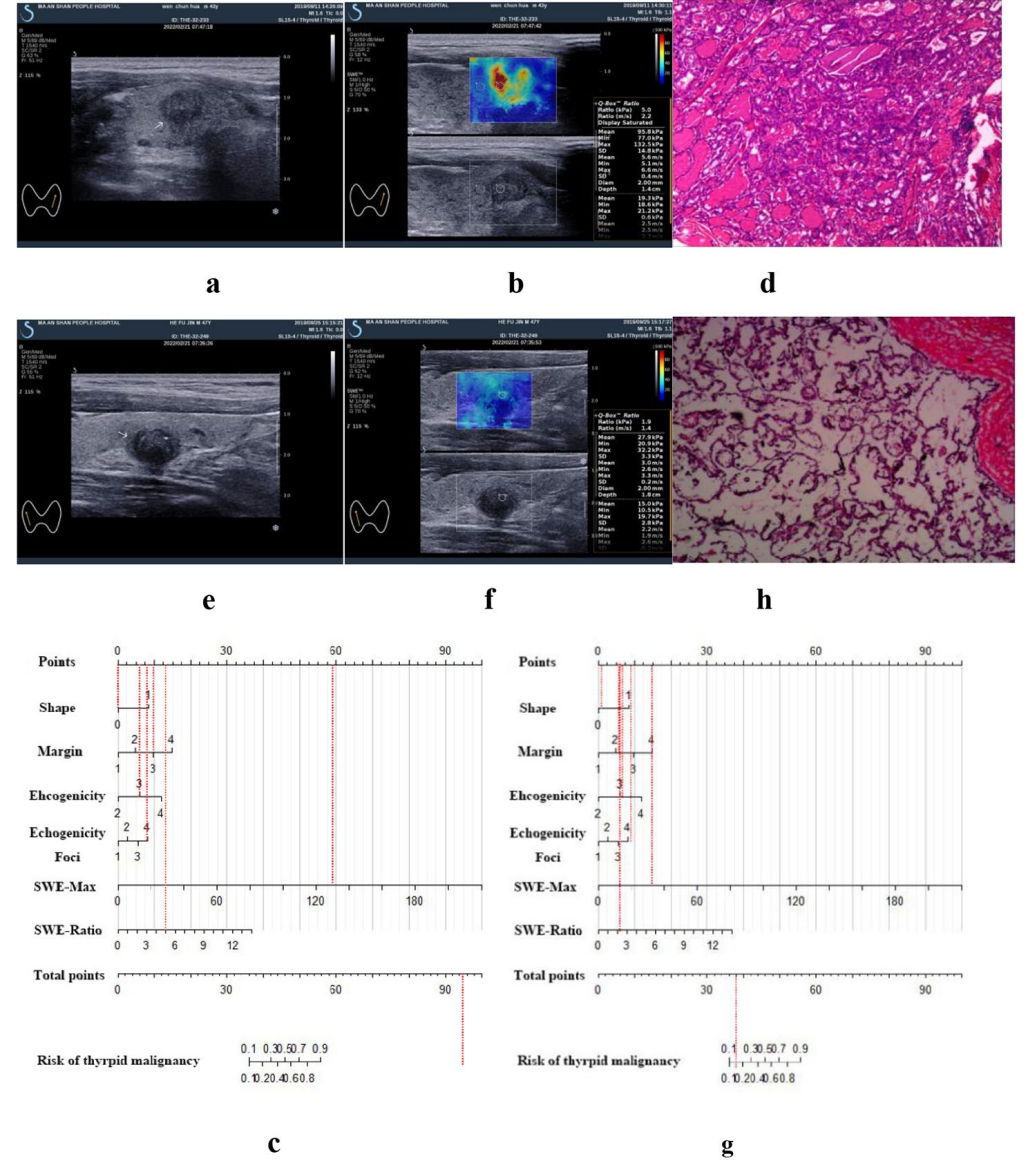
Example of the nomogram in use. **(a), (b)** Patient one with thyroid nodule (white arrow) with irregular margin, wider-than-tall shape, hypoechoic, punctate echogenic foci, SWE-Max of 132.5 kPa, and SWE-ratio of 5.0. **(c)** Risk of malignancy calculated by nomogram was more than 90%. (**d**, ×40) Pathological result confirms papillary thyroid carcinoma. **(e),(f)** Patient two with thyroid nodule (white arrow) with ill-defined margin, wider-than-tall shape, hypoechoic, punctate echogenic foci, SWE-Max of 32.2 kPa, and SWE-Ratio of 1.9. **(g)** Risk of less than 12%. (**h**, ×40) Pathological result demonstrates nodular goiter with cystic degeneration

For example, patient one had a nodule with irregular margins, wider-than-tall, hypoechoic, and punctate echogenic foci (Fig. 3a). In addition, the SWE-Max value was 132.5 kPa, and the SWE-ratio was 5.0 (Fig. 3b). The proposed nomogram value was calculated at more than 99% (Fig. 3c). Pathology proves that it is a papillary thyroid carcinoma (Fig. 3d, h&e, ×40). Patient two had a nodule with ill-defined margins, wider-than-tall shape, hypoechoic, and punctate echogenic foci (Fig. 3e). In addition, the SWE-max value was 32.2 kPa, and the SWE-ratio was 1.9 (Fig. 3f). The proposed nomogram showed a risk of less than 12%. (Fig. 3g). Pathology confirmed it was a nodular goiter with fibrosis (Fig. 3h, h & e, ×40).

## Discussion

Current assessment tools used to assess the risk of malignancy of thyroid nodules provide inconsistent results and are prone to observer variation. In addition, these tools are based solely on imaging or clinical features, thus further limiting their prediction accuracy. Therefore, this study aimed to develop a novel nomogram that integrates grayscale US characteristics, two-dimensional real-time shear wave elastography (SWE) measurements, and patient serum indices to characterize thyroid nodules.

This study used multiple logistic regression to analyze the current mainstream academic views. The results showed that the shape, margin, echogenicity, echogenic foci in grayscale US characteristics, and the values of SWE-Max and SWE-ratio were independent risk factors for thyroid cancer. Among them, the composition in grayscale US features was not an independent risk factor, which may be related to the included cases; there were no cystic or almost completely cystic cases. At present, simple cystic nodules are considered benign and do not need treatment [22]. Only three cases of cavernous nodules had pathological results. The other four grayscale US features are consistent in differentiating thyroid nodules. According to previous relevant studies, the elevated blood TSH level in patients with thyroid nodules is associated with an increased risk of thyroid cancer [23, 24]. TSH has no significance in the diagnosis of benign and malignant thyroid nodules in this study, which may be related to the number of samples in this study. SWE imaging is a new imaging technology developed in recent years. The American Thyroid Association (ATA), the American Association of Clinical Endocrinologists (AACE), the American Society of Endocrinology (ACE), and the Italian Association of Clinical Endocrinologists (AME) have recognized this method. It is recommended as an important means to evaluate the nature of thyroid nodules. Compared with quasi-static elastic imaging and strain ratio imaging, two-dimensional real-time SWE imaging provides a color-coded map of regional elastic value, quantitatively measures tissue hardness in kPa, and improves the positive predictive value. Papillary thyroid carcinoma mainly comprises diffuse and scattered sandy calcifications and collagen in papillary proliferative thyroid epithelial cells, tissues, stromal tissues, and hardened fibrous matrices. Studies [25, 26] have confirmed that the Young’s modulus varies in different thyroid tissues; the young’s modulus of normal thyroid tissues, cysts, thyroid adenomas, nodular goiters, and thyroid papillary carcinoma increased in turn. This study found that SWE-Max and SWE-ratio were independent risk factors for predicting thyroid cancer, which was consistent with Bardet S et al.’s [27] study. The European Federation of ultrasound (EFSUM) and the World Federation of ultrasound (WFUMB) released clinical application guidelines or an expert consensus of pre- and post-elastic imaging, affirming its value in examining benign and malignant thyroid nodules.

benign and malignant thyroid nodules. These guidelines clearly show that the applications of elastic imaging in evaluating thyroid nodules cannot replace conventional US but can be used as an auxiliary approach. In this study, a nomogram was constructed by combining grayscale US features with real-time SWE imaging to predict the risk of thyroid cancer. After internal verification by bootstrap and self-sampling, the C-index was 0.979 with high accuracy; the combination of the two improves the diagnostic efficiency. Compared to traditional TI-RADS, the model developed in this study can accurately determine the risk value of thyroid nodules rather than the interval range. Compared with the nomogram model constructed using grayscale ultrasound alone by other authors [28], the nomogram model constructed by combining grayscale ultrasound and shear wave elastography in this study showed good discrimination, with an AUC of 0.976 and good calibration.

### Limitations

There were some shortcomings in this study. First, this is a single-center study with a small sample size. Thus, we need to expand the sample size to improve the accuracy of the nomogram. Second, US-FNAB may lead to wrong puncture results. Third, the nomogram model passed an internal verification, but it also needs to pass an external verification to clarify the model’s abilities.

## Conclusions

In conclusion, the nomogram developed in this study represents a significant step forward in the diagnosis of thyroid cancer. Our novel nomogram integrated grayscale US characteristics and SWE measurements and provided a more accurate and comprehensive risk assessment for thyroid cancer. Compared with the traditional TI-RADS system, our proposed nomogram provides a simpler tool for the risk stratification of thyroid nodules. This innovation has the potential to improve the accuracy of thyroid cancer diagnosis and could ultimately lead to better patient outcomes.

## Data Availability

The datasets used and/or analyzed during the current study are available from the corresponding author upon reasonable request.
